# Classification of Odor-Derived Electroantennograms with Machine Learning

**DOI:** 10.1093/iob/obaf038

**Published:** 2025-11-03

**Authors:** Joshua Swore, Melanie Anderson, Marissa Dominguez, Tom Daniel, Jeff Riffell

**Affiliations:** Department of Biology, University of Washington, Seattle, WA 98195, USA; Department of Biology, University of Washington, Seattle, WA 98195, USA; Department of Biology, University of Washington, Seattle, WA 98195, USA; Department of Biology, University of Washington, Seattle, WA 98195, USA; Washington Research Foundation, Seattle, WA 98102, USA; Department of Biology, University of Washington, Seattle, WA 98195, USA

## Abstract

Insects have a keen ability to detect numerous odors in their environment. These odors, known as volatile organic compounds (VOCs), provide the insect with information about food, predators, and mates that may be in the area and are detected by olfactory receptors expressed by sensory neurons on the antennae. When VOCs are transduced by the olfactory sensory neurons, the antennal electrical potential dynamically changes, causing a local field potential (LFP) response to occur. Research has used the LFP amplitude for determining VOC concentration, but only recently have antennal LFPs been posited to be able to be used for VOC discrimination and identification. To close this gap, we use the time-series response of the antenna to odors as well as principal components of these responses to capture the characteristics of the LFP response, including waveform dynamics, intensity, slope, and duration. We use antennae of the *Manduca sexta* moth to record LFPs generated in response to Floral and disease associated VOC’s. Using machine learning approaches (support vector machines and random forests) trained on the LFP responses, we were able to predict and classify individual VOCs across a range of concentrations, as well as complex mixtures that elicited a given LFP waveform from an excised antenna. These results demonstrate that antennal olfactory responses can be used for the classification of differing VOC features, including concentration, identity, and duration, and have implications for diverse chemical sensing applications, such as search-and-rescue, the presence of agricultural pests, and the presence of human disease.

## Introduction

Detection of specific odors is vital for many tasks, including search dogs finding trapped people in disaster scenarios, detecting carbon monoxide and other dangerous gases in your home, and insects rely on their sense of smell to locate food and mates across large distances (Riffell et al. 2008). Engineered sensors and chemical analytical systems, such as metal oxide sensors and gas chromatography, have proven to be capable of accurately identifying chemicals, but are often large, cumbersome, and have slow response times (Andersson et al. 2015; Hirata et al. 2021). In contrast, when it comes to rapid and accurate odor detection, the canine nose is often considered the gold standard in odor recognition (Hirata et al. 2021). However, there are obvious cost and ethical considerations when using the canine olfactory system for chemical detection. The insect antenna is also a highly tuned and sensitive sensor that can be easily interfaced with electronic hardware and can rapidly detect chemicals present in the environment (Park and Baker 2002; Myrick et al. 2008; Anderson et al. 2020). In this work, we show that a live insect antenna can be used as a chemical sensor capable of classification of differing VOC features, including concentration, identity, and duration.

The *Manduca sexta* moth, which we use in this study, is a large insect species that uses its antennae to detect odors and search for food and mates (Hildebrand et al. 1992). The *M. sexta* moth has been a traditional model for neurodevelopment, neuroanatomy, and behavioral and functional studies (Hildebrand et al. 1992). With its abundant and sensitive olfactory neurons located in its antennae (Shields and Hildebrand 2001a,b) and accessible neurons in its brain, it has been an important model of olfactory research for the past 60 years (Gaurav and Ranjan 2024).

Over the last 20 years, the use of insect olfactory tissues for chemical detection has received growing interest (Myrick et al. 2009; Gaurav and Ranjan 2024). These studies have focused on either electrophysiological recording from the insect antennae, or from olfactory neurons located in the insect brain, with both approaches having strengths and weaknesses. Although olfactory neurons in the insect brain are sensitive and respond rapidly to changes in odor intensity, this process requires extensive surgery on the insect host which can limit its sensing time (Byers et al. 2013). Alternatively, insect antennae will continue to produce signals in response to odors for hours when removed (Park and Baker 2002; Anderson et al. 2020) and an experimenter can quantify their response to odor as a local field potential (LFP), which is a sum of excited receptor potentials on the antenna.

LFP have provided researchers with a direct electrical measure of information being communicated across nervous tissues, including those from the sensory periphery and the brain. Accessing this code and decoding these signals using machine learning (ML) has been an approach used for many biomedical implications. Results from these studies have provided information relevant to developing brain-computer interfaces and driving the movements of prosthetics (Kennedy et al. 2004a,b; Jackson and Hall 2016). Furthermore, LFPs from the sensory periphery can be used to detect explosives (Saha et al. 2020) and have been utilized to sense odors and guide unmanned vehicles (Anderson et al. 2019, 2020).

Recently, ML has been applied to the field of insect olfaction for odorant classification by molecular cues. Because the number of odorants able to be sensed by insects is so vast, ML techniques such as support vector machines (SVM), random forest (RF), and Naive Bayesian have been used to narrow down this number to a manageable amount for tasks such as identifying new pleasant smelling insect repellents (Kowalewski et al. 2024) or identifying orphan odorant receptor agonists (Kepchia et al. 2019; Jabeen et al. 2021). However, using ML methods on LFPs generated from excised antennae has been demonstrated only by one other group (Neta et al. 2023). Outside of insect olfaction, ML methods have been applied to classification tasks on LFPs generated by the human brain: electroencephalogram (EEG), showing that SVM and RF classifiers work well for detecting various emotions (Bhardwaj et al. 2015), and detecting brain disorders in newborns (Chen et al. 2014).

LFPs are generated by the electrical response of tissue within a sampled region. Examples of these recordings come from the brain cortex using an EEG, the heart via an electrocardiogram, or the antenna of an insect with an electroantennogram (EAG). In the case of the EAG, the LFP captures the voltage response of a collection of sensory neurons within the antenna and can be considered an evoked response via stimulation of an odor. When the odor binds to the receptors expressed by olfactory neurons, an action potential is generated. The sum of all action potentials generated by the activated neurons returns the overall voltage response of the antenna, usually appearing as a wave.

The EAG LFP contains both spatial and temporal information that can be leveraged for odor classification, analogous to the classification tasks involved with the EEG and EKG. Historically, studies have used the amplitude of the EAG wave to classify odors, with some odors evoking larger LFP amplitudes than others. This previous work has shown that it is possible that LFP amplitudes from a single antenna, or multiple antennae, can successfully classify odors (Park and Baker 2002; Myrick et al. 2009). However, there is a growing recognition that aspects of the LFP waveform itself can provide odor-specific recognition and improve classification (Neta et al. 2023). The insect antenna has diverse types of olfactory sensory neurons—each type is sensitive to different odors—that are often expressed in different regions of the antenna (Shields and Hildebrand 2001a). Moreover, the responses of these sensory neurons can also differ in the temporal dynamics of their responses (Raman et al. 2010). Together, the spatial and temporal heterogeneity of the antenna’s olfactory response could provide additional dimensions for odor classification beyond merely the EAG amplitude. A recent study used ML approaches to examine antennal LFP characteristics for odor classification and showed that classification improved compared to previous methods (Neta et al. 2023). However, it remained unclear how well the classification performed under conditions where the odors were similar to each other, or when embedded in a complex chemical background.

Here, using the *M. sexta* moth antenna, our study examines ML approaches for odor classification. Specifically, we explore the capabilities of 2 ML models, the SVM and RF algorithms, to discriminate LFPs produced by antennae. We investigate the classification performance under different stimulus conditions, including the effects of concentration, constituent vs. mixtures, and chemical structure similarities. Together, our study shows that the temporal features of the LFP of the EAG provide greater information for odor classification tasks compared to merely using the signal amplitude. This work has implications for the use of biohybrid chemical sensors capable of chemical detection and differentiation for diverse applications such as search-and-rescue, detection of agricultural pests, and even identification of human disease by breath volatiles.

## Methods

### Animals

All animals used in the study were males, maintained in a *M. sexta* colony by the Department of Biology at the University of Washington. Only male moths were used to control for sex-dependent odor sensitivity (Große-Wilde et al. 2010). Moths were allowed to eclose in cages with 12 h light/dark cycles. All moths were obtained for experiments on the day of use no more than 2 days post eclosion, during which they were food-deprived. A single antenna each from 15 moths were used for the experiments.

### Odor preparation

A panel of 8 odors, including, benzaldehyde, benzyl alcohol, 1-octen-3-ol, limonene, linalool, ylang-ylang, rose oil, and lemon oil were used for the study. All odors and mixtures were selected for their existence within the scent profiles of flowers known to attract the hawkmoth (Knudsen et al. 2004). All odors were purchased from Sigma–Aldrich. Odors were diluted in mineral oil at 1:1000 (1:1k), with select odors also diluted at a lower and higher concentration, 1:10000 (1:10k) and 1:100. Mineral oil elicits little to no response in the antennae and is used as a control for some of the tests shown in this study. A volume of 10 uL of the diluted odor was placed onto a strip of filter paper and placed into a prepared glass Pasteur pipette cartridge. The cartridges were sealed at the tip with teflon tape and sealed at the base with luers for ease of replacement and insertion into the odor delivery system for EAG experiments.

### Electroantennogram

**Fig. 1:**
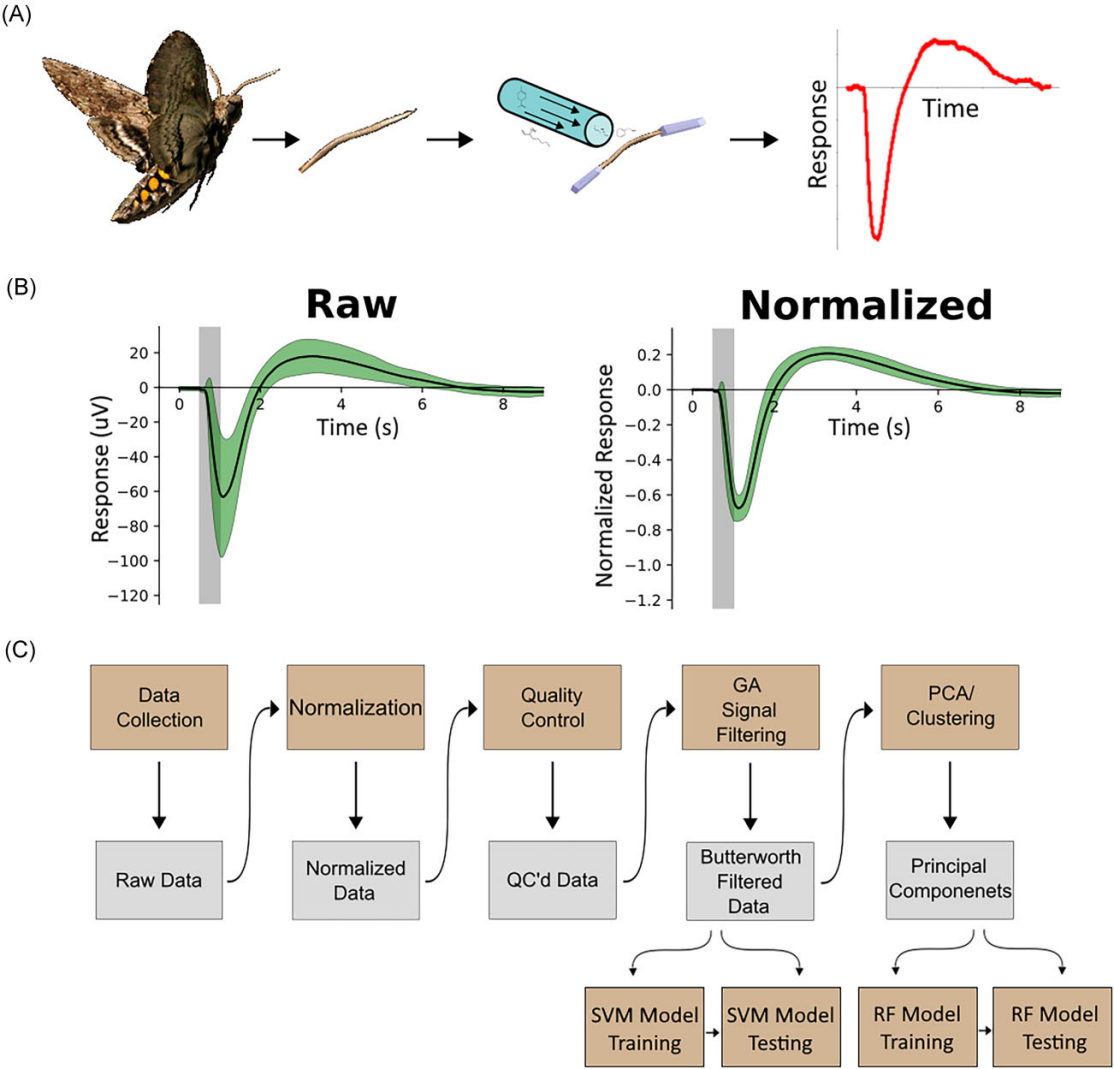
Experimental set-up, data processing, and Pipeline. (A) Experimental set-up. An excised *M. sexta* antenna is suspended between electrodes and exposed to volatile organic compounds (VOCs). The local field response of the entire antenna is recorded. (B) Signal normalization. Antennae responses were calibrated with ylang-ylang; responses to VOCs were normalized to the maximum and minimum of the ylang-ylang response. This technique resulted in reduced variance, indicated by the narrower shaded region in the normalized response plot, across experiments for each VOC tested. (C) Workflow. Data were collected, normalized, and passed through quality control where waves containing irrelevant frequencies were removed. A genetic algorithm (GA) was used to tune the parameters of a butterworth filter for signal filtering. The filtered time-series data was used in the SVM while principal components analysis (PCA) was used to produce dimensionally reduced components for the RF classifier.

Single site EAGs were conducted by suspending a single antenna excised from *Manduca* between 2 silver chloride electrodes. Electrodes were encased in glass capillaries filled with a 50/50 saline/electrogel. The base of the antenna, where the cut for excision occurred, was used for the recording electrode while the tip of the antennae was used as the reference. The antenna was exposed to a constant flow of neutral air that pulled across and away from the antenna via a vacuum funnel. Voltage recordings began 10 s prior to the delivery of the odor via a solenoid valve into neutral air. The solenoid valve was opened for 500 ms and repeated every 30 s to acquire 3 successive voltage responses to the odor. The order of odor delivery was randomized but the lowest to highest concentration of odor pattern was always used. For each antenna, all odorants were tested twice (6 total responses per odor per antenna), with ylang-ylang tested at the beginning, middle, and end of the antenna trial to account for any decreased responsiveness over time. Voltage changes in response to odor were measured on molecular devices Pclamp software (Fig. 1A).

### Data pipeline

Data were first collected via EAG. Following data collection files were converted from Axon Instruments’ Axon Binary Format (abf) format to csv files using custom python3 scripts and the pyABF package (Harden 2022). Individual waves were extracted and normalized to ylang-ylang where the minimum and maximum values from ylang-ylang were used in a standard min max normalization (Fig. 1B). Normalized extracted waves then underwent quality control by performing a fast Fourier transformation, finding the power spectral densities and then removing recordings in which unexpected frequencies were present. Data were then signal filtered offline with a butterworth filter in which the parameters were set via optimization with a GA. Signal filtered data was then used to train and test the SVM classifier. PCA was also performed on the signal filtered data. The first 5 principal components (PCs) were used in training and testing of the RF model (Fig. 1C).

### Data normalizing/standardizing

Data were normalized to the minimum and maximum values of the EAG induced by the ylang-ylang odor. Antenna were exposed to 1:1k ylang-ylang at the beginning, middle, and end of each test of an antenna. Ylang-ylang responses closest in time to the antennae recording of an experimental odor were used as the standard for normalization to account for any overall decrease in responsiveness over time. By calibrating the antenna to ylang-ylang and calculating the proportional response for each odorant, we were able to reduce the variance in the EAG response time across antennas (Fig. 1B). This process allows for a more accurate analysis in our ML models.

### Signal filtering and genetic algorithm for parameter tuning

An offline butterworth filter was applied to all data. The parameters for the butterworth filter were optimized for classification using a GA. This was chosen because GAs perform faster than an exhaustive grid search and are more accurate than a random grid search (Alibrahim and Ludwig 2021). A custom GA was written using python and the distributed evolutionary algorithms in python (DEAP) framework (De Rainville et al. 2012). Each individual in the GA contained 3 genes corresponding to 3 parameters for the butterworth filter (low cut: 0–1, high cut: 1.001–5, and order: 1–4). Each individual in the population was assigned a random value for each gene within the stated ranges. A total of 50% of the dataset was used for parameter optimization. A total of 100 individuals applied their parameters on the data and the PCs were calculated. Following the calculation of PCs, fitness was evaluated by calculating the difference between interclass variance and intraclass variance via Fisher’s discriminant ratio. To reduce the intraclass variance and maximize the interclass variance within PC space, we rewarded low variance within a class and high variance between different classes, minimizing the overlap between classes and creating greater separation.

### Machine learning

We used 2 supervised ML models, SVM and RF, to predict the odorant presented to the insect antenna. During preliminary testing, the unsupervised training model K-nearest neighbors and the supervised training model logistic regression were both tested, but neither performed as well as the SVM or RF models. SVM and RF are less computationally expensive and do not require as large of datasets as neural networks (NNs), therefore providing an optimal starting point for determining the capability of odor discrimination using these ML methods on single antenna LFPs. Using ML methods for odor discrimination on single antenna, LFPs has only been demonstrated by one other group (Neta et al. 2023), but other studies have shown that the SVM and RF classifiers work well for classification of LFPs from EEGs (Chen et al. 2014; Bhardwaj et al. 2015).

Optimization of both models included a random and also an exhaustive grid search. The random grid search was initially used in a broad parameter space to limit the space and reduce the computational time necessary to conduct an exhaustive grid search. Data was split 70/30 into training and testing sets with splits performed by antenna (done to prevent a classifier being tested on an antenna it had seen during training). The SVM model was trained and tested with the 5 s time series sample that included 250 ms prior to stimulation and 4750 ms following stimulation. Time series data was chosen based on preliminary testing that suggested this duration resulted in more accurate results over shorter durations. The RF model used the first 5 PCs which included a minimum of 90% of the variance seen in the dataset. Validation of the model included robust testing via 100 different splits of the data and calculating the balanced accuracy of each split. The mean balanced accuracy across the 100 splits of the dataset was used to assess model performance.

### Visualization

To visualize the results from our models, for each of the test scenarios, we show the following:

1. Mean voltage responses to the odorants used for the test.
2. Projection of the first 2 PCs including shaded 95% confidence intervals indicating where the mean of each class is located. Some constituents separate and cluster independently of the mixture. Overlap indicates similarity in wave responses.
3. Violin plots showing mean accuracies from 100 splits of the dataset indicate the models’ ability to correctly predict which odor was presented to the *M. sexta* antenna. Dashed lines represent the expected accuracy from random guess.
4. Confusion matrices showing performance in predicting individual odorants. The scores indicate the percentage the odor was predicted. High scores along the diagonal contribute to a high mean accuracy in the model.

## Results

### Limonene vs. control

**Fig. 2:**
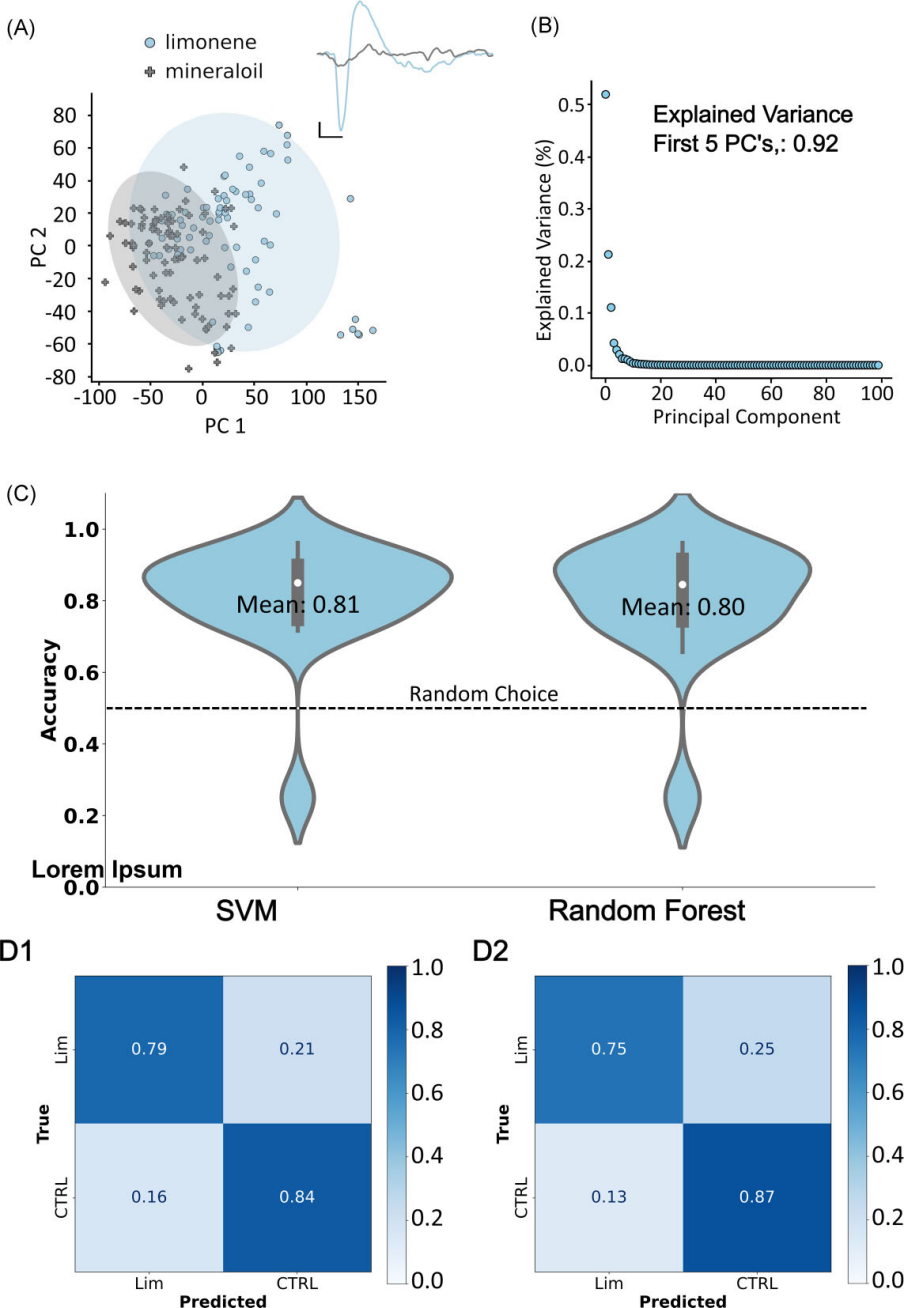
Discrimination of limonene from mineral oil. (A) Projection of the first 2 PCs with shaded 95% confidence intervals. (inset) Antennal responses to limonene (Lim) and mineral oil (CTRL). (B) PCs and their explained variance. The first 5 PCs contained 92% of the explained variance within the data set and were used for the RF model. (C) Average accuracy results for the SVM and RF models. The SVM classifier achieved 81% accuracy from the time series data, while the RF achieved 80% accuracy with the first 5 PCs. The dashed line represents the expected accuracy from random guess. (D) Confusion matrices for the SVM and RF models. In both models, limonene was correctly predicted $>$70%.

Limonene is a VOC found in abundance within a variety of citrus plants (IARC 1976). As insects navigate and explore the environment they may come across traces of this VOC. We first chose to explore *M. sextas* ability to detect limonene and then use ML to discriminate between traces of limonene and mineral oil offline. Mineral oil elicits little to no response in the antennae and is used as a dilutant for all the odors in this study. EAG LFPs from the insect antennae were recorded in parallel to the application of a 1:1k dilution of limonene in mineral oil (Fig. 2A inset). A depolarization followed by recovery was visible in EAG LFPs induced by limonene. In contrast, when mineral oil was applied, only small variations, uncorrelated with the delivery of mineral oil were recorded, consistent with noise seen in EAGs. Following the recording of EAGs, 100 PCs of the time series dataset were calculated and the first 2 components were used for visualization (Fig. 2A). Color-coded confidence ellipses were calculated and displayed to locate the true mean of each VOC within PC space. Both mineral oil and limonene EAGs are clustered separately though some overlap of confidence ellipses is likely due to extremely small waves induced by limonene and noise within EAGs during mineral oil application. The first 5 PCs explain 92% of the variance within our dataset of limonene and mineral oil. These 5 PCs were used for the training and testing of the RF classifier, while the first 5 s of the time series data, a total of 5000 features, were used for the SVM classifier.

Mean accuracies for each model after 100 splits of the data into training and testing sets are displayed in Fig. 2C. Both models performed similarly. The SVM classifier achieved a mean accuracy of 0.81 and the random forest classifier (RF) achieved a mean accuracy of 0.80. The confusion matrix reveals a sensitivity for limonene of 0.79 via SVM classification (Fig. 2D1) and 0.75 via the RF model (Fig. 2D2).

McNemar’s test was performed on the SVM and RF results, resulting in a *P*-value of 1.0 (Table 1). This indicates that there was not a significant difference in the performance of the 2 models when discriminating between limonene and mineral oil.

**Table 1:** McNemar’s test for RF and SVM on limonene vs. control

		SVM	
		Incorrect	Correct
RF	Incorrect	11	7
	Correct	6	59

### Mixture vs. constituent discrimination

**Fig. 3:**
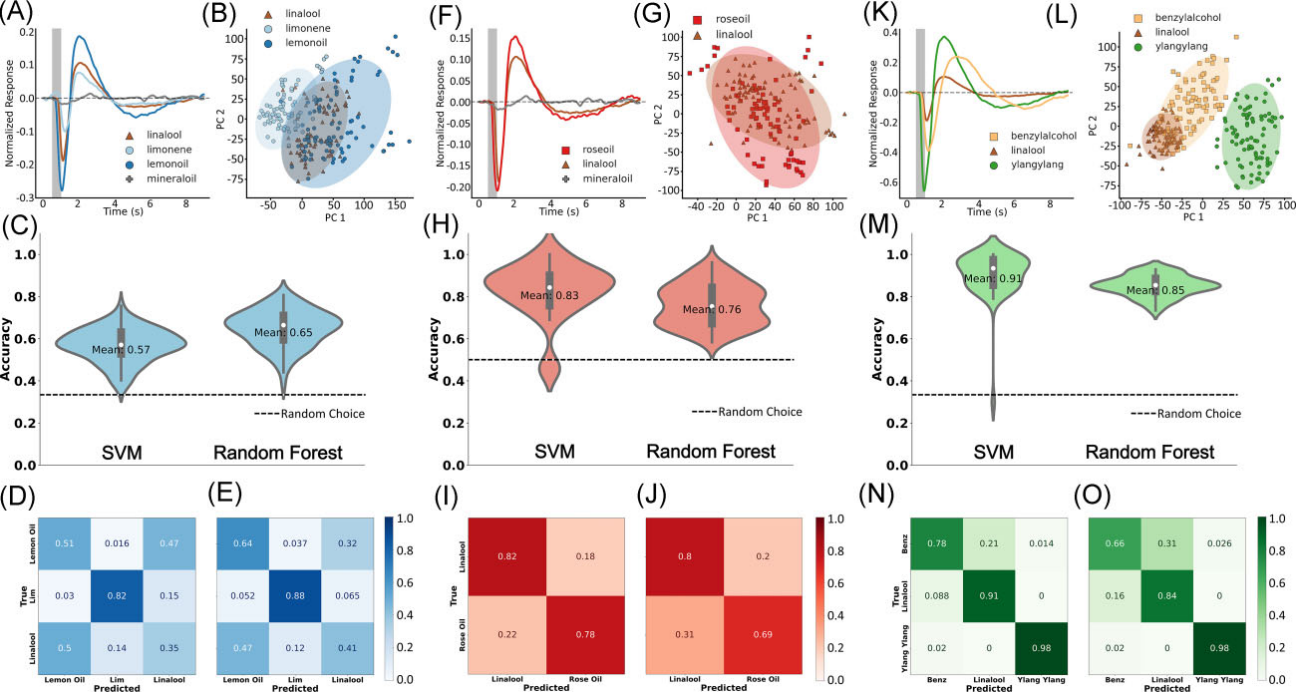
Discrimination of mixtures and constituents. (A, F, K) Mean antennal responses to linalool, limonene (lim), lemon oil, rose oil, Benzylalcohol, and Ylang-ylang. (B, G, K) Projection of the first 2 PCs with shaded 95% confidence intervals. (C, H, M) Mean accuracies for the SVM and RF models. The dashed lines represent the expected accuracy from random guess. (D, E, I, J, N, O) Confusion matrices for the SVM and RF models.

Though we were able to discriminate limonene from the control with some accuracy, limonene is only a constituent of the naturally occurring essential oils and other complex odors. For instance, limonene makes up approximately 70% of the lemon oil mixture (Gök et al. 2015; Kvittingen et al. 2021). Linalool is also a constituent of both ylang-ylang and rose oil. With this in mind, we asked if EAG LFPs recorded from the antennae could be used to discriminate the constituent from a complex mixture using SVM or RF ML models. EAG LFPs from 15 Moths (*N*=1593 total waves) responding to limonene, lemon oil, linalool, rose oil, and ylang-ylang were acquired, each displaying generally unique waveforms distinct from other odorants (Fig. 3A, F, K). We then set out to discriminate a single mixture (i.e., lemon oil, rose oil, or ylang-ylang) from the constituent (limonene or linalool).

#### Lemon oil, limonene, linalool

EAG LFPs elicited from stimulation by lemon oil, limonene, and linalool were notably different. Lemon oil consistently induced the largest of the 3 waveforms. Linalool induced the second largest voltage responses and limonene the smallest (Fig. 3A). Each response was generally unique to each odorant though projection of the first 2 PCs demonstrates that linalool and lemon oil responses produce overlapping clusters. The cluster of points associated with limonene had little overlap with the clusters of linalool and lemon oil (Fig. 3B). The shaded 95% confidence ellipses indicate where the mean of each cluster is likely located with minimal overlap.

We used 2 models for classification, a SVM and a RF. The voltage responses during the first 5 s following the onset of stimulation were used for classification with the SVM classifier. This allowed the SVM to use 5000 features for classification. The RF classifier utilized the first 5 PCs for classification striking a balance between accuracy and computational efficiency. These components contained 99% of the variance within the data set (data not shown). Mean accuracies for SVM and RF models were 57 and 65%, respectively (Fig. 3C). Both performed better than random guess indicated by the dashed line in Fig. 3C. The sensitivity, or ability to identify EAGS induced by a specific odor, for limonene was high in both models (82 and 88% respectively) (Fig. 3D, E). Lemon oil and linalool were difficult to discriminate with both classifiers indicated by incorrect predictions in the confusion matrices.

#### Rose oil vs. linalool

Rose oil and linalool produced similar EAG LFPs, though rose oil was capable of producing a stronger voltage response. We also observed a slight delay in the onset of the voltage response to linalool (Fig. 3F). The similarities of voltage responses between odorants were supported by the projection of the first 2 PCs, which resulted in an overlap of clustering points. The overlap of the 95% confidence ellipses indicates that the mean of linalool is likely within the cluster of rose oil (Fig. 3G).

We initially thought the overlap of clusters could make the accurate classification of linalool and rose oil difficult. Despite this overlap, discrimination of rose oil- and linalool-evoked LFPs occurred with some success. Classification accuracies were better than random (50%). A mean accuracy following 100 splits of the data set of 83% for SVM and 76% for RF were observed (Fig. 3H). The confusion matrix revealed a higher sensitivity to linalool in both models. Linalool was correctly predicted at a rate of 82% in the SVM model and 80% in the RF Model. Rose oil was correctly predicted at a rate of 78% in the SVM model and 69% in the RF Model (Fig. 3I, J).

#### Ylang-ylang vs. linalool vs. benzylalcohol

Ylang-ylang induced the largest voltage responses among all odorants tested. A subpanel of odor stimuli that included ylang-ylang, benzylalcohol, and linalool were the stimuli that indeed produced the largest responses. Benzylalcohol also produced large responses with a longer response in the positive direction. Linalool responses were shorter and less intense than both ylang-ylang and benzylalcohol (Fig. 3K). The projection of the first 2 components elicited a completely distinct cluster composed of ylang-ylang. Most points associated with either benzylalcohol or linalool clustered as separate classes, but with some overlap, indicating similarities between the 2 classes of EAG LFPs (Fig. 3L). Classification by SVM resulted in 91% mean accuracy from 100 splits of the data. The RF model resulted in a mean accuracy of 85%. Both models performed well above random guess (Fig. 3M). The confusion matrix of both models resulted in high accuracies along the diagonal indicating a strong ability to accurately predict individual odorants in the panel. In fact, ylang-ylang was correctly predicted 98% of the time in both models (Fig. 3N, O). The RF model had some difficulty predicting benzylalcohol in the RF model but still performed better than a random guess (66% vs. 50%) when deciding against linalool (Fig. 3O).

### Discrimination of floral mixtures

**Fig. 4:**
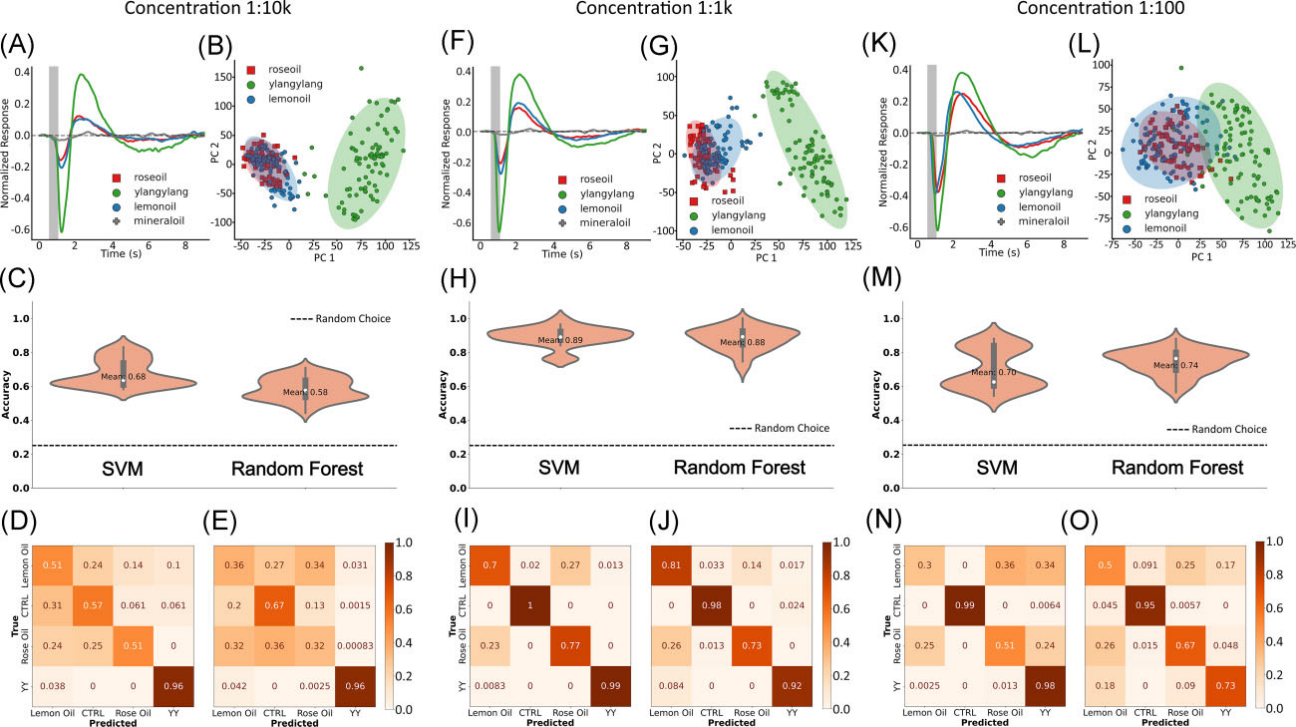
Discrimination of mixtures and concentrations. (A, F, K) Mean antennal responses to rose oil, ylang-ylang, lemon oil, and mineral oil (CTRL) at 3 concentrations: 1:10k, 1:1k, and 1:100 dilution in mineral oil. (B, G, K) Projection of the first 2 PCs with shaded 95% confidence intervals. (C, H, M) Mean accuracies for the SVM and RF models. The dashed line represents the expected accuracy from random guess. (D, E, I, J, N, O) Confusion matrices for the SVM and RF models.

We next asked if each floral mixture, composed of tens to hundreds of molecules, could be discriminated using SVM or RF models. The concentration of a given odor mixture will not be constant in the environment, so we chose to explore discrimination of odors at 3 different concentrations: 1:10k dilution, 1:1k dilution, and 1:100 dilution in mineral oil. Initially, each concentration was compared independently, as seen in Fig. 4. Fifteen moths were tested, for a total of *N* = 1074 waves for the 1:10k dilutions, *N* = 1083 waves for the 1:1k dilutions, and *N* = 1062 waves for the 1:100 dilutions.

At all concentrations, EAG LFPs elicited from ylang-ylang were larger than that of rose and lemon oils, as shown in the representative traces (Fig. 4A, F, K). Projection of the first 2 PCs supports this as ylang-ylang clusters on its own with minimal overlap (Fig. 4B, G, L). Rose and lemon oils, however, induced very similar voltage responses across all concentrations tested. EAG LFPs show similarities in the projection of the first 2 PCs, demonstrating a low variance between EAG LFPs produced by rose and lemon oils (Fig. 4A, B, F, G, K, L). Only the 1:1k dilution produces enough variance between rose and lemon oils for the clusters in PC space to begin separating (Fig. 4G).

Classification accuracies for 1:10k, 1:1k, and 1:100 dilutions were 68, 89, and 70% using the SVM classifier (Fig. 4C, H, M left) and 58, 88, 74% for the RF classifier, respectively (Fig. 4C, H, M right). At low concentrations (1:10k dilution), sensitivity of both models were severely reduced. The SVM classifier was able to correctly predict ylang-ylang but had some difficulty predicting rose oil, lemon oil, and the control (Fig. 4D). The RF classifier could not distinguish between lemon oil and rose oil, only accurately predicting ylang-ylang (Fig. 4E).

A similar pattern was observed at high concentration (1:100 dilution). The SVM classification accurately predicted both ylang-ylang and the control but had difficulty distinguishing between rose and lemon oils (Fig. 4N). The RF classifier performed slightly better accurately predicting the control and distinguishing between lemon oil, rose oil, and ylang-ylang better than random guess (Fig. 4O).

Discrimination of mixtures was best at the medium concentration (1:1000 dilution). The SVM classifier was nearly perfect at correctly predicting ylang-ylang and the control. Distinguishing between rose and lemon oils occurred with 77 and 70% accuracy, both better than random guess (Fig. 4I). The RF classifier was better at predicting lemon oil than the SVM with 81% accuracy and performed similarly in regards to predicting rose oil. The RF classifier was accurate at predicting ylang-ylang and the control (Fig. 4J).

### Effects of odor concentration on discrimination

**Fig. 5:**
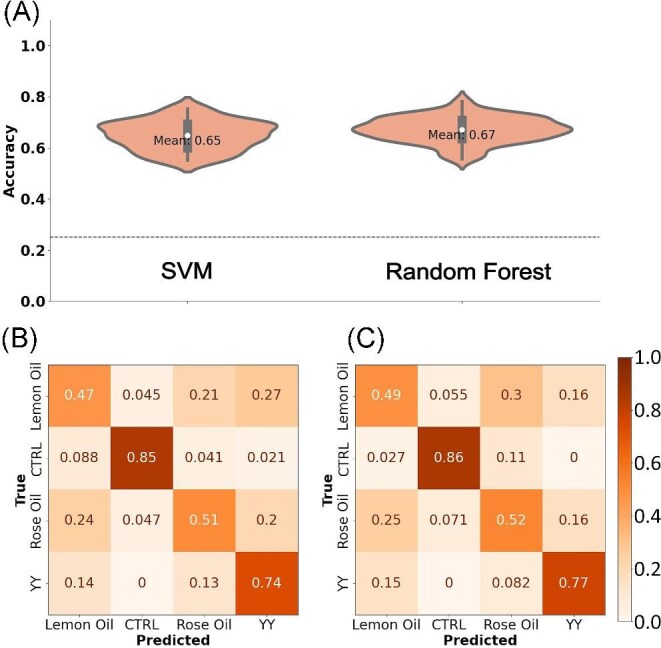
Discrimination of mixtures at multiple concentrations. (A) Mean accuracies for the SVM and RF models. Both models achieved 71% accuracy in the ability to correctly predict which odor was presented to the antenna. The dashed line represents the expected accuracy from random guess. (B, C) Confusion matrices for the SVM and RF models. Both SVM and RF have high sensitivity to correctly identify ylang-ylang at any concentration.

Prior to this, we have only tested our models on a single concentration. We next sought to test both models in their ability to classify odors when multiple concentrations were present in the data. In this case, the model could be presented with an EAG LFP produced by an odor at any of 3 different concentrations and was then required to identify the odor. This was done to better simulate the varying odor concentrations present in a plume as an insect navigates its environment. All LFPs were normalized to the response to only the 1:1k dilution of ylang-ylang. Again, the first 5 PCs were used for classification. The SVM model achieves an average accuracy of 67%, while the RF model achieves an accuracy of 65% (Fig. 5A). Even when presented with multiple concentrations of each odor, the models were able to identify ylang-ylang and the mineral oil (solvent) control most of the time. Lemon oil and rose oil, however, were often mistaken for each other (Fig. 5B, C).

### Chemical functional group discrimination: linalool, benzyl alcohol, 1-octen-3-ol

**Fig. 6:**
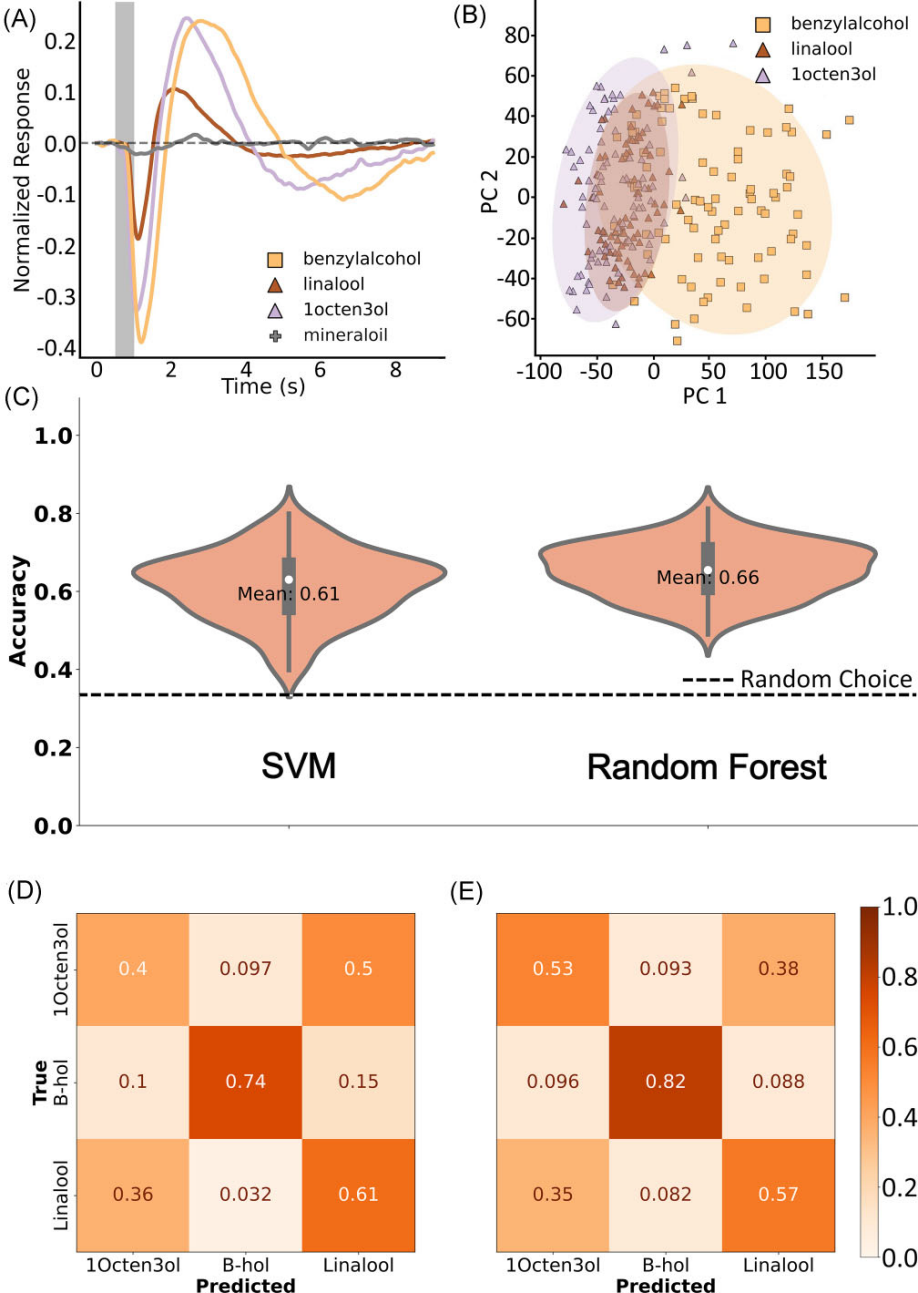
Discrimination of alcohols. (A) Mean antennal responses to benzylalcohol (B-hol), linalool, and 1Octen3ol. (B) Projection of the first 2 PCs with shaded 95% confidence intervals. Overlap indicates similarity in wave responses. (C) Mean accuracies for the SVM and RF models. The dashed line represents the expected accuracy from random guess. (D) Confusion matrices for the SVM and RF models.

We last asked if our models could distinguish odorants with similar chemical structures based on the EAG LFP waveforms. The stimulus panel contained 3 odorants with an alcohol ($-$OH) group. The shared alcohol group could contribute to similar binding activities with the antennal odorant receptors, producing similar features within the LFP responses. Responses from 15 moths were used resulting in *N* = 257 total waves. The mean LFP responses of linalool and 1-octen-3-ol were very similar. Only benzyl alcohol produced a response that was larger and dissimilar from linalool and 1-octen-3ol (Fig. 6A). Projection of the first 2 PCs showed the variability between benzyl alcohol and the other VOCs. EAGs produced by linalool and 1-octen-3-ol cluster together with very little separation. However, EAG LFPs elicited by benzyl alcohol were distinctly clustered from the other odorants (Fig. 6B).

Classification of alcohols achieved 61% accuracy via SVM classification and 66% classification via RF, both better than random (Fig. 6C). Observation of the confusion matrix unsurprisingly reveals that both classifiers were able to distinguish benzyl alcohol but had difficulty distinguishing between linalool and 1-octen-3-ol (Fig. 6D, E).

## Discussion

### Biohybrid classifiers

Here in this study, we present analyses of the EAG LFP to understand some of the current strengths and limitations of the insect antenna as a biohybrid discriminator when combined with ML models. Moth antennae are capable of detecting and producing an electrical response unique to each of our tested odorants. In most cases, we found that both SVM and RF models are capable of discriminating and identifying odorants based on single-channel EAG waveforms. Our study demonstrates the high accuracy both SVM and RF models can achieve when the antenna is presented with the right concentration and select odor subset. This could be useful when prospective odorants are known and the user has the ability to control the concentration and number of prospective odorants. However, in some cases, we found that structurally similar odorants may lead to low classifier performance. Nonetheless, this work shows that the insect antenna provides a fast mechanism to detect chemicals in the air, much faster than that of current metal oxide detectors.

### Model performance

Our models performed best when there were large differences between the tested stimuli. This was also shown in a recent study that used a locust antennae as a chemical sensor and RF as the model (Neta et al. 2023). In our current study, the PC analysis revealed cases where distinct separation between odor stimuli were observed. Models performed best when classes exhibited little to no overlap in the PCA space. These well-separated cases included comparisons such as ylang-ylang versus its constituents, ylang-ylang versus rose and lemon oils, mixtures at a 1:1000 dilution, and benzyl alcohol versus linalool and 1-octen-3-ol. Although PCA dimensionally reduces the data, our SVM classifier which was trained on the time-series data instead, performs similarly to the RF classifier which was trained on the first 5 PCs only. Future studies can explore if manually extracting features such as rise time, fall time, and time-to-peak could enhance model performance.

Linalool and benzyl alcohol are constituents of the complex odor that is the ylang-ylang essential oil. At lower concentrations, when linalool or benzyl alcohol binds with odorant receptors expressed on cognate olfactory neurons, they will only activate those specific sensory neurons. However, when ylang-ylang is presented to the *Manduca* antenna, both linalool and benzyl alcohol will bind to their respective receptors and simultaneously activating their populations of neurons. When recording the EAG LFPs, the sum of both populations rather than just a single population is recorded resulting in a larger, and perhaps distinct, response. Thus, the summed response will provide greater variance between ylang-ylang and its constituents. This increased variance makes it easier for the classification model to distinguish between ylang-ylang and its constituents.

Similarly, the chemical composition of ylang-ylang is different compared to rose and lemon oils. Therefore, the receptors and neural populations activated when presented with the odors will be distinct. Different neuronal populations will produce unique wave forms that have enough variance between the ylang-ylang, rose, and lemon oils, allowing for accurate odor classification.

The odor concentration also had a strong influence on classification. At the 1:1000 dilution, the projection of the first 2 PCs results in observable separation between the rose and lemon oil essential oils. However, at both lower and higher concentrations, this separation is not present. At low concentration, this is likely due to low neural activation and making the signal difficult to extract. While at high concentrations, the signal may be saturated due to limitations of our recording equipment or biologically by activation of neural populations and non-specific receptor binding eliciting waveforms that are not unique to either odor. Previous work using EAG LFP amplitudes from moth antennae and k-nearest neighbor classification models has shown accurate classification for distinct odors (Myrick et al. 2008), and more recent work using RF models of locust antennal responses has also shown accurate classification success (Neta et al. 2023). Our current study builds on this work by showing the interplay between odor concentration, similarity in chemical structure, and classification success when a cognate odorant is embedded in a complex mixture.

The process of normalizing to ylang-ylang yielded much better classification results than a min–max normalization in preliminary testing. Normalizing the responses to ylang-ylang altered the between-class variability but is acceptable within the scope of this study to demonstrate that RFs and SVMs could be used to identify the odors presented to a hawkmoth. In future studies, the method of normalization can be optimized for the context of the desired result. For instance, if the desired outcome is to use a drone equipped with antennal sensors to autonomously identify the source of a chemical leak within a disaster zone, the ML pipeline would have the flexibility to be built and optimized (normalization method included) for the detection of the desired chemical (natural gas, propane, etc.). Antennae used in this context could be calibrated by their response to ylang-ylang or a different compound to enhance discrimination. In the section, Effects of Odor Concentration on Discrimination, we show that the models do not show much of a decrease in performance when tested on multiple concentrations of odors even though all responses are normalized to a single concentration (1:1k) of ylang-ylang.

### Future scalability and prospects

In this study, we show that SVM and RF models are capable of discriminating and identifying odorants based on simple single-channel EAG LFPs. This classification could further be improved by combining these models with genetically modified antennae or with antennae from different species of insect (Myrick et al. 2008).

Recent studies have shown that using gene-editing to knock-out specific odorant receptors in *M. sexta* may alter the antennal response to certain odors (Fandino et al. 2019; Tom et al. 2024). Combining and comparing the responses from gene edited antennae and unedited antennae could enhance the discrimination capabilities of our ML classifiers. This could also be extended to antennae from other insect species that have differing sensitivities to certain odors (Myrick et al. 2009).

While this study focused on floral compounds and mixtures, *M. sexta* antennae have been shown to be responsive to a variety of compounds, including various ketones and aldehydes which are present on human breath (Shields and Hildebrand 2001b; Chen et al. 2021). When infected with an illness, such as COVID-19, the human metabolism causes these compounds to present in differing concentrations on the breath than in a healthy individual (Chen et al. 2021). *Manduca* antennae combined with ML models could be a useful, non-invasive, and near-instantaneous diagnostic tool to identify various illnesses in individuals.

Insect antennae have been shown to have greater sensitivity, responsiveness, and selectivity than commercially available portable chemical sensors (Anderson et al. 2020; Gaurav and Ranjan 2024). In this study, we show that applying ML models to LFPs from the *M. sexta* antennae allows the classification and discrimination of various floral compounds. Moreover, in contrast to recording from olfactory neurons in the brain, recording the LFPs from excised antennae allows for a simplified preparation process and repeatable, stable recordings while still resulting in the classification of odors using ML methods. The use of excised antennae could allow these methods to be used with odor-localizing robotic platforms such as drones (Anderson et al. 2019, 2020), where motor vibrations prohibit sensitive preparations such as antennal lobe recordings. Such robotic platforms could be used in diverse applications, such as search-and-rescue efforts and detection of harmful compounds. Here, our study provides motivation for the incorporation of real-time ML approaches on such robotic platforms (Anderson et al. 2020).
